# Effectiveness of a cognitive-behavioral therapy (CBT) manualized program for clinically anxious children: study protocol of a randomized controlled trial

**DOI:** 10.1186/1471-244X-12-16

**Published:** 2012-03-12

**Authors:** Mélou Jansen, Marleen MEM van Doorn, Anna Lichtwarck-Aschoff, Rowella CWM Kuijpers, Huub Theunissen, Mirjam Korte, José van Rossum, Annemiek Wauben, Isabela Granic

**Affiliations:** 1 Behavioural Science Institute, Radboud University Nijmegen, Montessorilaan 3, 6525 HR Nijmegen, The Netherlands; 2 Pro Persona Youth Arnhem, Klingelbeekseweg 19, 6812 DE Arnhem, The Netherlands; 3 Pro Persona Youth Nijmegen, Tarweweg 2, 6534 AM Nijmegen, The Netherlands; 4 Ambulatorium Nijmegen, Montessorilaan 10, 6525 HR Nijmegen, The Netherlands

**Keywords:** Anxiety, Cognitive-behavior therapy, Alliance, Parenting, Randomized controlled trial

## Abstract

**Background:**

In the Netherlands, the prevalence of anxiety disorders is 20%; and children with anxiety are at increased risk for psychopathology throughout adulthood. Recently, a revised version of a cognitive behavioral therapy manualized program called 'Thinking + Doing = Daring' (TDD) was developed for children between 8 and 12 years old with an anxiety disorder. The main aim of this project is to conduct a Randomized Controlled Trial (RCT) to evaluate the effectiveness of TDD.

**Methods/Design:**

The CBT program will be tested with a RCT with 120 clinically anxious children (8-12 years old) referred to one of three mental health care agencies. Children will be randomly assigned to the experimental (*N *= 60, TDD) or to the control condition (*N *= 60, treatment as usual). The primary outcome measure will be the child's anxiety symptoms level. Secondary outcome measures will be externalizing (e.g. aggression) and internalizing problems (e.g. depression). Two potential mediators of change will be examined in the current study: therapeutic alliance and parenting. Mother and child in both the experimental and control condition will be surveyed at baseline, post treatment and after 6 and 12 months (follow-up). It is hypothesized that children in the experimental condition will show a stronger decrease in anxiety symptoms compared to children that receive treatment as usual. Moreover, we expect that a strong therapeutic alliance and decreases in parental control and rejection will contribute to treatment success.

**Discussion:**

Early treatment for anxiety problems has the potential to not only result in anxiety reductions, but also to prevent future problems such as substance abuse and psychopathology throughout adulthood. Our results will be immediately relevant to practice, since we are partnering with 'real world' community agencies. If the CBT program proves more effective than treatment as usual, it could be implemented in community mental health care agencies across the Netherlands and beyond. Moreover, it has the potential to make treatment in these community settings shorter, more efficient and therefore cost-effective. Trial registration: Nederlands Trial Register NTR2967

## Background

Anxiety disorders are one of the most common types of psychopathology during childhood [1]. In Europe, 12-20% of children experience anxiety [2]. More than 12% of the children is diagnosed with an anxiety disorder. In the Netherlands the prevalence of anxiety disorders is even higher than 20% [3,4]. Various forms of anxiety disorders exist. The most common anxiety disorders in childhood are the specific phobia, social anxiety disorder, separation anxiety and generalized anxiety [5].

A characteristic feature of all anxiety disorders in children is the preoccupation with danger. Although the type of stimulus eliciting fear may change over time due to developmental transformations, anxiety and fear are a chronic problem. In an attempt to cope with the perceived threat of the outside world children with anxiety disorders learn to avoid potentially threatening situations. As a result, the social and academic functioning of children with anxiety is jeopardized. Children with anxiety have fewer friends and receive lower grades at school [6]. Additionally, the importance of parental factors (e.g. genetic transmission, anxious modeling, over-controlling parenting style) in the etiology and maintenance of childhood anxiety is well established [7]. In turn, as a result of a child's anxiety family life can be impaired such that families with anxious children perceive more stress and participate less in social activities [6]. Finally, clinical levels of anxiety during childhood present great risks for future development, such as an increased risk for substance abuse and suicidality during adolescence [8] and higher rates of psychopathology and educational underachievement in adulthood [9,10]. Because of the high prevalence of anxiety in children and the detrimental effects on socio-emotional and academic functioning, which bear great challenges for future development, much research has been devoted to identifying effective interventions to target childhood anxiety.

Cognitive behavioral therapy (CBT) - with or without parental involvement - is consistently being identified as the most effective treatment for childhood anxiety. A recent meta-analysis among 24 studies found an overall posttreatment remission rate of anxiety disorders of 55.4% and showed a mean overall effect size of CBT is .86 [11]. All CBT treatments share similar ingredients such as exposure to anxious situations, cognitive restructuring of dysfunctional thoughts, relaxation before and during anxious situations and positive self-talk. In the Dutch context a protocollized CBT treatment for anxious children and adolescents called 'Thinking + Doing = Daring' (TDD) has been developed by Bögels [12] based on these principles. Importantly, the intervention integrates parents by teaching them how to communicate with their child about anxious situations and how to motivate and support their child in overcoming its fear. Also the parent's own fears and anxieties are being discussed. The treatment consists of twelve weekly sessions with the child and three sessions with the parents. Three months after therapy, a follow-up session takes place.

In a study by Bodden, Bögels and colleagues [13] the effectiveness of the TDD-treatment was tested with a randomized controlled trial including three different conditions. Children were between eight and 17 years old and either received the TTD (individual CBT with little parental involvement), a family CBT or were put on the waitlist for eight weeks. Some of the families in the waitlist condition received the treatment after eight weeks. These post waitlist results were included in the effect calculation. At post-treatment 41% of the children was free of all anxiety disorders and 56% was free of their primary anxiety diagnosis. All waitlist children still had anxiety disorders after the waitlist period. At three-month follow up these percentages were 52% and 67% respectively. Concerning the difference between the TTD and the family CBT, the study found better treatment outcomes for the TDD (56% recovered from anxiety) compared to the family CBT (28% recovered from anxiety). The effect size for the TDD was 1.39 and 1.03 for the family CBT (as measured with the parent version of the dutch version of the Screen for Child Anxiety Related Emotional Disorders (SCARED-NL). In families where parents had an anxiety disorder, children also benefited more from the TDD (46% recovered from anxiety at post treatment) compared to family CBT (19% recovered from anxiety at post treatment). Also, more dropouts were found in the family CBT-condition (19%) than in the TDD (3%). In sum, the effect sizes of the TDD program are promising and based on this study's results the TDD, that is an individual CBT with little parental involvement, seems to be more beneficial than a family CBT. The child and therapist manuals of the TDD are published including assessments and treatment integrity forms, and the program is now widely used in the Netherlands.

The primary aim of this study is to replicate and extend the findings of the Bodden et al study [13] in a randomized controlled trial. In contrast to the Bodden et al study [13] two conditions will be used: the experimental condition which will consist of the TDD program and the control condition which will consist of treatment as usual (TAU). The ultimate proof of the effectiveness of a treatment program is when it exceeds the effects of the treatment that families and children normally receive. Furthermore, to test the long-term effects of treatment six-month and 12-month follow up assessments will be conducted. Importantly, this study will take place in the real-world context, where co-morbidity is the rule rather than the exception [14]. In this way the study's results can be generalized to the context where the intervention may eventually be delivered, that is in mental health institutions in the Netherlands.

Finally, despite the promising results of CBT so far, variability in treatment outcomes remain. Not all children with anxiety profit from therapy. It is not clear why some children fail to show improvement in therapy, since we have little understanding of the underlying mechanisms and processes of change. Randomized controlled trials inform us *if *a certain interventions works but they do not tell us by what mechanism, information that is essential in order to further improve and tailor intervention efforts. Two potential mediators of change will be examined in the current study: therapeutic alliance and parenting.

Many researchers have confirmed the importance of alliance in adult therapy. Stronger therapeutic alliance predicts better outcome [15,16]. However, the role of alliance in child-therapy has received little attention so far and results are mixed. Kazdin [17] found that strong therapeutic alliance predicted more improvement in the child. However, in a study by Liber [18] alliance and treatment outcome were only moderately related. In two studies by Kendall [19,20] no significant association between alliance and treatment outcome were found. In the adult literature, alliance at one month after treatment has started is usually used to predict treatment outcomes [16]. However, alliance is likely to fluctuate across the treatment period. In order to test when alliance best predicts treatment outcomes, alliance will be assessed at multiple time points: one and two months after treatment has started and at post treatment.

The second potential mediator is parenting. Childhood anxiety is more common in families with anxious parents, suggesting a familial transmission of anxiety [21]. Numerous studies in previous years have focused on the influence of family interactions in the development, maintenance, and improvement of childhood anxiety (e.g. [22,23]) and found several potential parenting behaviors influencing childhood anxiety. The dimensions *rejection *and *control *received a great amount of consideration in the parenting literature (e.g. [24-26]), but with different definitions and mixed results. Recently, McLoad, Wood and Weisz [27] conducted a meta-analysis on both dimensions and found that both rejection (small effect) and control (medium effect) were associated with childhood anxiety. If treatment for childhood anxiety is effective, parent-child interactions are likely to change. In the current study, we will track changes in the dimensions rejection and control.

### Aim and hypotheses

The primary aim of the study is to evaluate the effectiveness of the CBT-program Thinking, Doing and Daring (TDD) by comparing it to treatment as usual (TAU). Our primary outcome, children's anxiety, will be assessed through parent reports and children's self-reports. To measure long-term effects of treatment, follow-up assessments at three month, six month and one year follow up will be conducted. The second aim is to analyze whether there are secondary positive outcomes beside recovery of anxiety. Secondary outcomes (e.g. depression, aggression) will be assessed through parent and teacher reports. The third aim is to analyze the potential mediating influence of alliance and parenting on positive treatment outcomes.

More specifically, we expect that a) children in the experimental condition will have significantly less anxiety symptoms after treatment and at the follow-up measurements than children who received treatment as usual, b) children who recover from an anxiety disorder will also show a significant reduction in secondary problem behavior (e.g. depression, aggression), c) children who form a strong alliance with their therapist will have less anxiety symptoms than children who form a less strong alliance, d) parent-child dyads for those children who improve through therapy will show less parental control and rejection after treatment than at the start of treatment.

## Methods/Design

### Trial design

**Figure 1:**
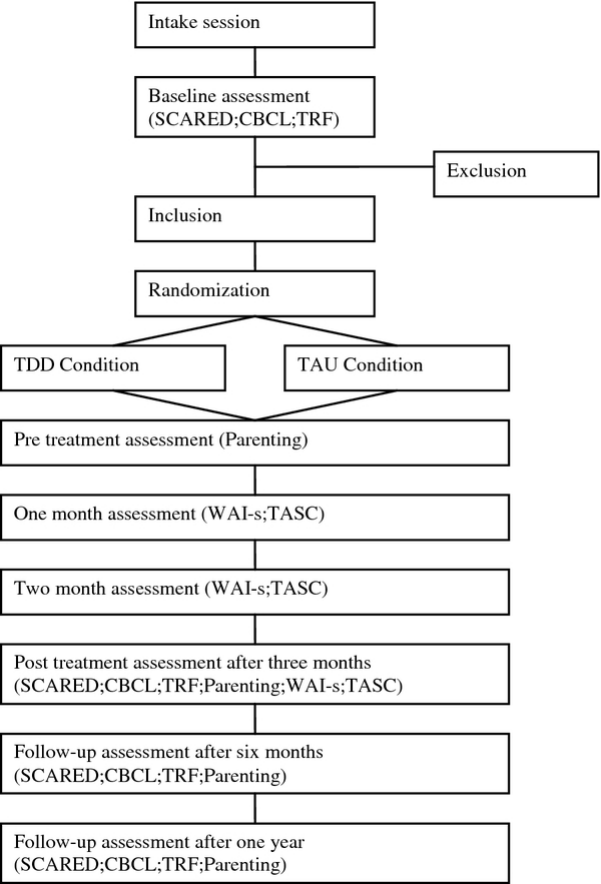
**Flow chart of the phases of the randomized trial**.

The effectiveness of the TDD program will be tested in a RCT in which three Dutch community mental health agencies (Pro Persona Youth in Nijmegen and Arnhem and the Ambulatorium Nijmegen) will participate. A total of 120 clinically anxious children (8 - 12 years old) and their parents will participate in this study after filling in a consent form (see Figure 1 for the study design). The participants will be randomly allocated to the experimental (TDD, *N *= 60) or control condition (TAU, *N *= 60). The TDD consists of twelve weekly sessions with the child and three sessions with the parents. The treatment is supported with child, parent and therapist manuals. Children in the control group will receive the treatment that is usually delivered in those agencies. This means that these children will receive the treatment that the therapist considers to be the most effective treatment for that particular child. Baseline assessments, post-treatment, a six-month follow-up and a one year follow-up will be conducted among children, mothers and therapists and through direct observations (see Table 1).

**Table 1:** Measurements collected at each wave

	Measurement waves					
	**Baseline/pre treatment**	**1 month**	**2 month**	**Post treatment**	**6 month follow-up**	**1 year follow-up**

SCARED-NL	X			x	x	x

CBCL	X			x	x	x

TRF	X			x	x	x

TASC		x	x	x		

WAI-s		x	x	x		

Parenting	X			x	x	x

To compensate for their time filling in the research questionnaires, parents will receive a financial contribution and children will receive little gifts (such as pens, stickers and small candy). Ethical approval has been granted by the ethical committee of the Faculty of Social Sciences at the Radboud University Nijmegen (ECG16122010).

### Participants

Child-participants between the age of eight to twelve years will be recruited at one of the agencies. As part of the usual intake procedure families are asked to fill in standard questionnaires to collect information for diagnostic assessment and indication criteria. When a child is between eight and twelve years old additional questionnaires will be administered. These are: the Child Behavior Checklist (CBCL), the Teacher Report Form (TRF) and the Screen for Child Anxiety Related Emotional Disorders (SCARED) and are meant to assess children's level of anxiety and to determine the presence of additional problems. Inclusion criteria are a score above the clinical cut-off on either the child or parent version of the SCARED-NL total scale or one of the following subscales: generalized anxiety, social anxiety, separation anxiety and panic disorder. Exclusion criteria are autism, post traumatic stress syndrome, specific anxiety disorder, obsessive-compulsive disorder and an IQ below 80. Medication is allowed and will be assessed during the measurement waves.

Families with an eligible child will be informed about the project by one of the researchers and will be asked to participate. When a family agrees to participate, they fill in an informed consent form. Families will be randomly allocated to the TDD or TAU condition using a blocked randomization scheme (block size 4). Randomization will be done by an independent researcher at the Behavioural Science Institute.

### Therapists

All therapists (n = 16) participating in this study work at one of the three agencies. Two therapists are mental health workers and 14 therapists are graduated psychologists of which 12 have a registration as a Health Care psychologist ('GZ-psycholoog'). Seven therapists are between 25 and 40 years old. Five therapists are between 40 and 55 years old, and four therapists are older. Years of experience of the therapists varies between three and 27 years. At each institution the therapists will be randomly assigned to the experimental or control condition. The therapists in the experimental condition will participate in a two-day training in the protocol provided by the author of the manualized protocol, Susan Bögels. In this training the protocol will be explained and practiced on each other during exercises and role-plays. The therapist in the control condition will receive no extra training.

### Intervention

#### Experimental condition

The experimental condition (TDD) consists of twelve weekly sessions with the child and three sessions with the parents. In the first session the rationale behind cognitive behavior therapy will be explained to the child and its parents. During the second session the child learns to identify his anxious thoughts and how to generate alternative thoughts. Challenging anxious thoughts is also discussed. Relaxation and mind-distraction is practiced in the third session. In the same week as the third session, a parent session is taking place where the parent will be made aware of the influence of their own fear on the behavior of the child. Parents are also taught how to support their child in every step it takes facing his fear. In the fourth session children make a hierarchy of anxious situations they want to face during therapy. Also, self rewarding is explained in this session. Exposure to the anxious situations starts in session five and lasts until session ten. In the same week as session six, the second parent session is scheduled. Parents will be taught how to support their child during exposure tasks. Their thoughts about their child will be discussed and challenged. In the seventh and eighth session challenging thoughts will be illustrated by doing an experiment in which the child learns to restructure his thoughts. In session nine the child will be motivated to talk more with his parents about its fear. The parents are also motivated to communicate more with their child about anxiety and with each other about their parenting styles. It will be explained how spouses can support each other. A summary of the learned skills during therapy is given in session ten. The last exposure task is a game or a quest. How the child should deal with set backs is discussed in session eleven. In the last session, session twelve, the therapy ends by giving the child a certificate. How the child can deal with new anxious situations without the therapist is also discussed. Each month after this session the therapists will make a phone call to stimulate the child to keep using the learned skills. Three months after session twelve a follow-up session is held with the child and his/her parents. The therapy, the change in behavior of the child and the learned skills will be evaluated. Each therapy will be supported by a therapist manual, and child and parent workbooks. Within three months this treatment will be completed.

#### Control condition

Children and families in the control condition (TAU) will receive treatment that is being normally given at each institution during the period of three months. There are no restrictions for this condition and we will track the types of interventions provided. The therapist will decide what the treatment should be. Usually therapy for anxious children consist of various sessions in which therapists use different parts of protocols or from their own clinical experience, mostly based on cognitive restructuring, exposure tasks and relaxation tasks with the child and a few sessions with the parents. Sometimes other techniques such as EMDR (Eye Movement Desensitization and Reprocessing) or Mindfulness are used.

### Data collection

In order to compare our findings with those of Bodden et al [13] we will use similar instruments for both child and mother. The anticipated flow of data collection is graphically shown in Table 1. All therapy sessions will be audio-taped. For treatment integrity, three sessions per client will be randomly selected and coded. We will use coding measures of Bögels to track treatment integrity.

#### Outcomes

The primary outcome, anxiety symptom level, will be measured with the SCARED-NL [28], a screening instrument for children. The child self-report (C) and parent-report (P) version consist of 69 identical items which differ only in the substitution of you/your child. Mother and child score each item on a 3-point scale ranging from 0 (never or almost never) to 2 (often). The SCARED-NL demonstrates good convergent and divergent validity compared with psychiatric diagnoses and/or structured psychiatric interviews [29,30].

Secondary outcome measures are depression and aggression which will be assessed with the Child Behaviour Checklist (CBCL; [31]) and the Teacher Rate Form (TRF; [32]). Both questionnaires describe a wide domain of internalizing and externalizing behavior problems of children. The CBCL consists of 113 items and the TRF consists of 118 items; ninety-three items are overlapping. Both mother and teacher are asked to rate each item on a 3-point scale ranging from 0 (does not apply to the child) to 2 (clearly or often). The checklist provides T-scores for internalizing and externalizing behavior. Both the CBCL and the TRF show satisfactory psychometric properties [33].

Therapeutic alliance between therapist and child will be measured with two alliance questionnaires. The therapist will fill in the Dutch translated version of the Working-Alliance-Inventory-Short Form (WAI-S; [34,35]). The WAI-S was developed to measure Bordin's three aspects of alliance: the bond, agreement of tasks-, and goals [36,37]. The scale consists of 12 items on a 7-point scale. Research has demonstrated the reliability and validity of the scale [38,39].

The child will be asked to fill in the Dutch translated version of the Therapeutic Alliance Scale for Children (TASC-nl; [40]). The TASC-nl includes 12 items which have to be completed on a 4-point scale ranging from 1 (not at all) to 4 (very much). The TASC was designed specifically for the use with children and adolescents. Positive and negative aspects of the therapeutic alliance are measured. In previous research the TASC has demonstrated adequate internal consistency (a = .72 to.74) [40,41].

The possible mediator parenting will be assessed through observations of structured mother-child interactions [42,43], taking place in the homes of the families at a time convenient for them. Interactions will be videotaped and subsequently coded for the dimensions rejection and control. In the study the focus is on mothers, since they are in most cases the primary caregiver and we want to standardize across participants and measurement waves. A standardized paradigm will be used for observations of mother-child interactions. Mother and child will engage in three 5-minute episodes: (1) a competitive sports game on the Nintendo Wii console, (2) a discussion about something the child is anxious about in the coming week, and (3) a cooperative sports game on the Nintendo Wii console The digital video recordings will be coded using Noldus Observer XT. Three assistants will be trained to reliably code videos for the dimensions rejection and control. Assistants will be intensively trained to a minimum criterion of 75% agreement and 0.65 kappa using a frequency/sequence-based comparison and a criterion of 80% agreement using a duration/sequence based comparison. Recalibration training will be conducted to minimize coder drift. A second coder for reliability purposes will code 20% of all sessions. Coders will be blind to which sessions will be used to assess observer agreement and also blind to the condition and when in the treatment protocol the observations were collected (pre, post or follow-up).

### Sample size calculation

The study aims to assign 120 anxious children to the project. The children will be equally divided across both conditions. Power analysis (G-power) is based on a 3-month effect size of 1.0 taking into account a maximum of 20% attrition over time and loss of power due to multiple imputation. Sample sizes will be 60 families per condition (alpha < .05, power = .80). For testing the effect of the potential mediators, we do not need to run a RCT per se. However, as we are the first running a follow-up study on Bodden et al. [13], it is essential to establish whether we will obtain similar effect sizes. Hence, we can not estimate the variability in the mediators beforehand - as this has not been examined in these kinds of CBT treatments before - but previous work on other types of pathology [44] and the high effect size necessary to examine differences at all, provide us with confidence that our design and sample size is suitable. The sample sizes are large for observational treatment studies involving parent-child interactions [45]. Excellent observational studies with similar or smaller sample sizes have been published in top-tier journals. This is largely due to the fact that micro-coding in observational designs holds repeated measurements of individual variables [46].

### Statistical analyses

In accordance with the intent-to-treat philosophy, all children randomized to a condition will be included in the analyses to test the study hypotheses. Analyses will be conducted using Mplus, which is a statistical modeling program that has special features to deal with missing data and it allows analyses with complex data while taking into consideration the longitudinal character of the data. Regression analyses will be conducted to test whether children in the experimental condition (TDD) show a stronger decrease in anxiety symptoms than in the control condition (TAU). Also for the second aim of our study, namely testing whether secondary problem behavior (i.e. aggression, depression) decreases more in the experimental condition, regression analyses will be conducted. Third, to investigate the mediating role of alliance and parenting, mediation analyses will be performed in Mplus, using bootstrap methods.

## Discussion

The design of this study is a randomized controlled trial to test the effectiveness of the TDD program, developed by Susan Bögels, for eight to twelve years old children with anxiety. It is hypothesized that children that follow the TDD treatment will show a stronger decrease in anxiety symptoms compared to children that receive treatment as usual. Moreover, we expect that a strong therapeutic alliance and decreases in parental control and rejection will contribute to treatment success.

### Strengths and limitations

An important strength of this study is that we will use a control condition in which children will not be put on a waitlist, as many other RCTs do, but where children will receive treatment as usual. In this way it will be a stronger test of the effectiveness of the TDD program. Second, long term effects of the program will be examined with one year follow up assessments. Furthermore, the vast majority of RCT studies focuses solely on the effectiveness of the tested program, that is *if *a certain intervention works, but do not examine *how *the intervention works (i.e. the mediators of change). This study's aim is to understand why some children improve and others not by a) testing if alliance is responsible for therapeutic change, and b) testing if parental control and parental rejection mediate treatment outcome. A limitation of the study is that only mothers can participate. While there are several good reasons for this choice (e.g. mothers are more likely to spend time with their children and to participate in intervention and research programs), previous research has shown that mothers and fathers uniquely contribute to the development, maintenance and amelioration of children's anxiety [47]. Hence, future research should involve fathers in order to test differential effects of mothers and fathers.

### Implications for practice

Since all referred children between eight and twelve years old will be screened, anxiety will be recognized early in development. Early treatment for anxiety problems have the potential to prevent future problems, such as substance abuse and psychopathology throughout adulthood [8,9]. Further, this project aims to unravel some of the underlying mechanisms of treatment success of anxiety disorders in children. This will subsequently lead to improvement of care. These insights will be used for improving the protocols of this specific treatment. It will make treatment shorter, more efficient and therefore cost-effective. Finally, in the project we are partnering with "real world" community agencies. Therefore, our results will be immediately relevant to practice and there is potential for large-scale roll-out across the Netherlands.

## Competing interests

The authors declare that they have no competing interests.

## Authors' contributions

MJ and MvD are the two PhD candidates running the study primary in a period of 4 years. Both will closely work together in terms of data collection and coding. vD will focus on parenting and J will focus on therapeutic alliance. All other authors are supervisors, grant applicators and co-investigators. All authors read and approved the final manuscript.

## Pre-publication history

The pre-publication history for this paper can be accessed here:

http://www.biomedcentral.com/1471-244X/12/16/prepub
